# A frontal attention mechanism in the visual mismatch negativity

**DOI:** 10.1016/j.bbr.2015.07.022

**Published:** 2015-10-15

**Authors:** Craig Hedge, George Stothart, Jenna Todd Jones, Priscila Rojas Frías, Kristopher Lundy Magee, Jonathan C.W. Brooks

**Affiliations:** a School of Psychology, Cardiff University, UK; b School of Experimental Psychology, University of Bristol, UK; c Clinical Research and Imaging Centre, University of Bristol, UK

**Keywords:** Attention, Inferior frontal gyrus, Mismatch negativity, Visual attention, vMMN

## Abstract

Automatic detection of environmental change is a core component of attention. The mismatch negativity (MMN), an electrophysiological marker of this mechanism, has been studied prominently in the auditory domain, with cortical generators identified in temporal and frontal regions. Here, we combined electroencephalography (EEG) and functional magnetic resonance imaging (fMRI) to assess whether the underlying frontal regions associated with auditory change detection also play a role in visual change detection. Twenty healthy young adults completed a visual MMN task in separate EEG and fMRI sessions. Region of interest analyses were conducted on left and right middle frontal (MFG) and inferior frontal (IFG) gyri, i.e., the frontal areas identified as potential auditory MMN generators. A significant increase in activation was observed in the left IFG and MFG in response to blocks containing deviant stimuli. These findings suggest that a frontal mechanism is involved in the detection of change in the visual MMN. Our results support the notion that frontal mechanisms underlie attention switching, as measured via MMN, across multiple modalities.

## Highlights

• We examine frontal mechanisms underlying the visual mismatch negativity.
• EEG and fMRI activity was examined in respect to unattended oddball stimuli.
• Left inferior frontal gyrus was associated with changes in the stimuli.
• Our findings correspond to similarly implicated regions in the auditory domain.

## 1 Introduction

The Mismatch Negativity (MMN) is an electrophysiological response that reflects the automatic detection of change in the sensory environment, and is elicited by violating an established regularity in a sequence of sensory stimuli. Such violations can take the form of simple physical changes in the stimulus properties (e.g., [47]), abstract deviations in the relationships between stimuli (e.g., [3]), or a non- symmetrical stimulus in a sequence of symmetrical stimuli (e.g., [29]). Since its first description [42] it has become an established tool in the investigation of sensory processing and attention, and a marker of cognitive decline across a variety of conditions (see [43] for a review). After the initial focus on the auditory MMN (aMMN), there is now an established body of evidence for MMN in the visual modality, the vMMN (see [32,34,50,80] for reviews), as well as somatosensory (e.g., [30]) and olfactory modalities (e.g., [36]). Electrophysiological and functional imaging studies suggest a role for both frontal and sensory (temporal lobe) sources of the aMMN (see [10] for review); however, there is limited evidence to date that addresses this question in the vMMN. Thus, the aim of this study was to use both EEG and functional imaging to assess whether a frontal source contributes to the vMMN, which may indicate a multi-modal mechanism for the low-level detection of stimulus change.

Studies of the aMMN have implicated a role for the frontal lobe, with some of the earliest work from Näätänen and Michie [44] suggesting two distinct neural sources underlying the MMN: a superior temporal generator associated with comparison of incoming stimuli with memory traces of previous stimuli; and a frontal generator related to involuntary triggers of attention associated with a change in stimulus. Further work addressed the dissociation of the two proposed neural generators revealing a bilateral (although dominantly right-hemisphere) frontal source in response to infrequent changes in pitch or duration [22,11]. Dipole modeling studies have provided inconsistent evidence for a frontal generator, with most demonstrating that the aMMN can be accounted for by two dipoles located in the superior temporal gyrus (e.g., [58]) with the addition of small, but significant, increases in explained variance with the addition of sources in one or multiple frontal regions. These include the medial frontal gyrus [21], left, right, or bilateral inferior frontal gyrus (IFG; [11,55,59]), and anterior cingulate cortex [26]. fMRI and PET techniques have provided evidence for right IFG activation [39,46] left IFG [40] and bilateral IFG [56,83] following changes in the pitch of acoustic stimuli. Changes in the presentation duration of acoustic stimuli have been associated with increased activation in both the left [39] and right IFG, with some activation also seen bilaterally in the IFG and in regions of the lateral frontal cortex [14,57,59]. This apparent variability in the location of the frontal source may stem from the variations in the degree of attentional focus on the stimuli. Recent work using independent components analysis to examine the oscillatory characteristics of the aMMN has demonstrated that the strength of frontal source responses is modulated by the active or passive nature of a task, as well as stimulus complexity [37].

Given that the aMMN network appears to be comprised of two bilateral auditory cortex sources interacting with a frontal source, it is possible that the frontal source may be involved in the MMN response in other sensory modalities. Recent theoretical and empirical work in the context of the aMMN [19,20], and the vMMN [31,64,65], have discussed the interactions between frontal and sensory areas in the context of hierarchical predictive coding. In this account, the MMN reflects an error signal that is generated when a sensory input does not match a prediction for that input. Frontal mechanisms are thought to underlie the coding of the predicted representation [31,71], which then feeds back to sensory processing regions. Thus, frontal regions are strongly implicated in the vMMN, however, the location and nature of frontal mechanisms has yet to be unequivocally characterized in the literature. There is converging evidence from other paradigms that a frontal mechanism may be sensitive to changes in multiple modalities. Downar et al. [15] used fMRI to examine modality- specific and common networks underlying the passive detection of changes in sensory stimulation in visual, auditory and tactile modalities. Uni-modal activation was observed in visual, auditory, and somatosensory processing areas in respect to each modality, and multimodal activations were observed in a network including bilateral IFG and right insula. Kimura et al., used the spatial modeling technique, sLORETA, for scalp recorded EEG to dissociate any other sources of the vMMN from the visual N1. Frontal activation was found to be associated with the vMMN response in orbital and rectal gyri [33]. A vMMN study using emotional face stimuli implicated a prefrontal mechanism in healthy adults [8]. In another visual deviance detection paradigm, using fMRI to examine responses to infrequent visual stimuli during a visuo-motor tracking task of varying difficulty, activation was observed in the prefrontal cortex, albeit in more medial regions than the lateral activity reported by Kimura et al. [82]. Yucel et al.’s task is somewhat distinct from typical MMN designs in that the primary task has a relatively high cognitive load. This is likely to involve the recruitment of a set of regions which commonly show increased activation in the presence of increased task demands [5,18], as well as dual tasking [35]. These regions also overlap with those implicated as frontal MMN mechanisms, which may moderate the locus of activity observed. Elsewhere, Urakawa et al. [78] used MEG to examine visual deviance detection and demonstrated a large middle occipital gyrus response to infrequent stimuli that was followed by activation in the precuneus and right IFG in three out of eight participants. Finally, an fMRI study of visual change detection in adults with autistic spectrum disorders and healthy adults observed left lateralized frontal and occipital activity [84]. Overall there is a limited but growing body of evidence to suggest a role for frontal areas in visual deviance detection, though its functional role and location are as yet unclear. Specifically, the variation in the cognitive demands of the tasks used in the literature make it difficult to dissociate the mechanisms of low level change detection associated with the vMMN response from processes involved in the active identification of oddball stimuli. The aim of this study was to use the improved localisation of fMRI in conjunction with EEG to investigate the vMMN response to simple visual object change detection without the potential confounding effects of cognitive load.

One line of support is the overlap between the regions implicated as frontal sources for the aMMN and regions associated with the control of visual-spatial attention and cognitive control. In the attention networks framework of Posner and colleagues [53], developed primarily from work on visual-spatial attention, the left IFG has been associated with maintaining a state of alertness to incoming stimuli, and both left and right IFG with an executive attention network [16]. The right IFG has featured prominently in accounts of the mechanisms underlying cognitive control [1]. Recent findings suggest that subregions of right inferior frontal cortex perform distinct roles, with the more dorsal inferior frontal junction acting to detect cue changes, and the IFG supporting the consequent updating of a current action plan in response to these cues [25,79]. Mirroring the findings from aMMN paradigms, whilst a strong emphasis has been placed on the role of the right IFG in these tasks, left or bilateral IFG involvement has been implicated by imaging and lesion studies (e.g., [73,74], for review). A full account of the debate surrounding the role of the IFG in executive control is beyond the scope of this paper, and caution should be taken when drawing inferences about functions across paradigms, though this work highlights a role for this region more broadly in theories of attention. Moreover, the infrequent cues that required detection and behavior adaptation in the studies mentioned were visual, which suggests that if they underlie change detection in the aMMN, they may also be involved in the vMMN.

To examine the potential role of frontal mechanisms in the vMMN, we adapted a variant of a visual mismatch task that has been reported previously in the literature [63,69,77] to be used within a block design functional magnetic resonance imaging (fMRI) study. In addition to having participants perform the task in a separate EEG session, we counterbalanced the combination of visual stimuli used, to verify that any effects observed reflected attentional, rather than stimulus specific, mechanisms. Regions of interest for analysis were derived from the common frontal areas showing activation across 15 fMRI and PET studies examining the neural sources of the aMMN reviewed by Deouell [10].

## 2 Methods

### 2.1 Participants

Twenty healthy younger adults (aged 21–34, mean age 25.1 (±4.5), 7 males) took part in the study. Participants gave their informed written consent prior to participation in accordance to the Declaration of Helsinki, and the experiments were approved by the local Ethics Committee. Participants were recruited from the University of Bristol student population, had no known neurological or psychiatric disease, and declared themselves to have normal or corrected to normal vision, and self-reported as being right-hand dominant. Participants completed both an EEG and an MRI session. The order of the sessions was counterbalanced across participants.

### 2.2 Stimuli and procedure

Stimuli were presented using Presentation software version 12.2 (Neurobehavioral Systems, Inc.).

**Fig. 1:**
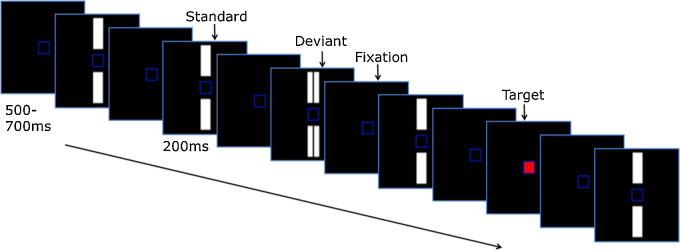
Stimuli used to elicit visual mismatch negativity. Standard and deviant stimuli type was counterbalanced across blocks.

The experimental design was identical for both EEG and fMRI. Using a paradigm adapted from [77], participants were instructed to fixate and attend exclusively to a small blue frame (1.3° × 1.3° visual angle) within a larger blue frame that stayed on-screen for the duration of the study (10.5° × 10.5°; see Fixation stimulus Fig. 1). Stimulus size in visual angle was equated for the fMRI and EEG sessions. Periodically, the centre of the blue frame turned red (see Target stimulus Fig. 1) and the participant was required to respond to this change as quickly as possible by pressing a hand-held button. Participants were instructed to ignore any other stimuli that appeared on-screen and focus solely on the fixation square and target stimuli. The target presentation was a rare event for which subjects would have to maintain a sharp attentional focus. Single white bars (3.9° × 1.2°) and double white bars (3.9° × 0.6° each) that were equal to the single white bars in total area and brightness were presented simultaneously above and below the central blue square. Single and double white bars were counterbalanced as standard and deviant stimuli; we henceforth refer to the combination of a double bar deviant with a single bar standard as combination A, and the combination of a single bar deviant with a double bar standard as combination B.

Stimuli were presented in 18 counterbalanced blocks. Eight blocks contained solely standard and target stimuli (we refer to these as ‘standard only’ blocks). Ten blocks contained standard, target and deviant stimuli. The presentation order of the 18 blocks was fully randomised. Each block lasted between 100 and 140 s, with rest periods of 15–25 s between blocks. The reason for the use of a block design, rather than an event-related design, is that MMN tasks require a relatively short inter-stimulus interval in order that subjects retain a strong sensory memory trace to the preceding stimulus. An event-related design would require a longer gap between consecutive stimuli, potentially significantly reducing the memory trace of the preceding stimulus. Note that previous fMRI studies of the MMN generators have also used block designs (e.g., [39,45]).

The standard, deviant and target stimuli were presented for 200 ms with a randomised inter-stimulus interval (ISI) of 500–700ms. Targets and deviants were presented in a pseudo- random sequence among the standards, such that at least two standards preceded each deviant. The ratio of standards:deviants:targets was 16:1:1, with 1248 standards, 78 deviants and 78 targets presented. In the blocks containing standards and targets only 1248 standards were presented and 78 targets. The order of the sessions (EEG and fMRI) was counterbalanced across participants, each session lasted approximately 36 min.

### 2.3 EEG recording

EEG signals were continuously recorded from 64 Ag/AgCl electrodes fitted on an elasticised cap in a standard 10–20 electrode layout using a common FCz reference. Signals were sampled at a rate of 1 kHz using a BrainAmp DC amplifier (Brain Products GmbH). Impedances were kept below 5 kΩ and all signals were online low-pass filtered at 250 Hz during recording. Recordings were analysed offline using Brain Electrical Source Analysis software version 5.3 (BESA GmbH). Artefacts including blinks and eye movements were corrected using BESA automatic artifact correction [2] and any remaining epochs containing artifact signals > ±100 uV were rejected. The rejection rate never exceeded 10% of trials for each participant and condition. Epochs from −100 to 600 ms were defined around stimulus onset, were baseline corrected using the pre-stimulus interval (−100 to 0 ms), and low-pass filtered at 40 Hz.

### 2.4 Electrode selection

Seven electrodes: O1, Oz, O2, PO9, PO10, PO7, and PO8 were selected for statistical analysis on the basis of existing reports on early visual evoked potentials (c.f., Stothart et al., 2012), and their values averaged to form an occipital region of interest. Examination of the mean spectral power across the scalp and grand average evoked responses re-referenced to a common average reference confirmed that the vMMN was primarily located in the occipital region and that the electrode selection was appropriate. Common average referenced plots of responses to standard and deviant stimuli across all 64 channels are presented in Supplementary information B.

### 2.5 Event Related Potential (ERP) analysis

For the ERP analysis only blocks containing the standard, target *and* deviant stimuli were examined. To measure the vMMN, the averaged response to standard stimuli at the selected electrodes was subtracted from that to the deviant stimuli to create a difference waveform separately for stimulus combinations A and B. Sequential one sample *t*-tests were then applied to the difference waveforms using the method outlined by Guthrie and Buchwald [24]. The consecutive time points necessary to indicate an epoch of significant difference between the responses to the standards and deviants were obtained from a simulation using an autocorrelation estimated from the data (34 for stimulus combination A and 18 for stimulus combination B). Time intervals with values of *p* < 0.05 that lasted for the required consecutive time-points or longer were accepted as epochs of significant difference. vMMN was defined as any negative deflection of the difference waveform following the P1 response that lasted for the necessary consecutive time-points. Mean difference waveform amplitudes were then calculated for each participant for the vMMN epoch.

### 2.6 MRI recording

Whole brain imaging data were acquired using a 3 tesla Siemens Magnetom Skyra MRI system. A magnetisation prepared rapid gradient echo (MPRAGE) pulse sequence was used to acquire high-resolution T1-weighted anatomical images of the whole brain with the following parameters: flip angle = 9**°**, FOV = 240 mm, matrix = 256 × 256, slice thickness = 0.9 mm, with TE/TI/TR = 2.25/800/1900 ms. To improve registration of functional imaging (EPI) data to the MNI template, fieldmaps were generated from a dual echo gradient echo sequence with flip angle = 60**°**, FOV=192 mm, matrix = 64 × 64, slice thickness = 3 mm, with TE1/TE2/TR = 4.92/7.38/520 ms. Functional imaging data were acquired using an EPI sequence and flip angle = 90**°**, FOV = 192 mm, matrix 64 × 64, slice thickness = 3 mm thickness (0.75 mm inter-slice gap) and 40 slices aligned to the AC–PC line, with TE/TR = 30/2500 ms. Total scanning time lasted approximately 45 min. The first two dummy images were discarded to allow for equilibration of the blood oxygenation level dependent (BOLD) signal.

### 2.7 MRI analysis

All data were processed and analysed using FMRIB Software Library 5.0.2.1 (http://fsl.fmrib.ox.ac.uk/fsl/fslwiki/). Prior to statistical analysis functional data were motion corrected using MCFLIRT [27], spatially smoothed with a 5 mm full-width half-maximum Gaussian kernel and temporal high pass filtered with cut-off at 200 s. Subsequently, each subjects’ functional images were linearly registered to their structural scan using a brain boundary registration (BBR) algorithm [23]. This registration used the computed fieldmap, EPI echo time and phase encoding direction, to estimate and unwarp the signal displacement induced in functional images by local magnetic field variation. The unwarped, linearly transformed EPI data were then registered to the 2 mm resolution MNI 152 standard brain, by non-linear registration of each subjects’ structural scan to the template by using FNIRT [66]. Particular care was taken to ensure that frontal regions, which suffer from the effects of magnetic susceptibility induced signal distortion [9] when imaged with echo-planar imaging (EPI) sequences, were accurately registered to the Montreal Neurological Institute (MNI) template brain. Explanatory variables were defined to account for activity due to baseline and targets, as well as standard and deviant stimuli from both stimuli combination A and B. The rest periods between blocks were used as a baseline. Group statistics were calculated using a paired *t*-test within a mixed effects general linear model with a cluster forming threshold *Z* > 2.3, and cluster corrected *p* value < 0.05 [81].

Estimated contrasts were compared at the higher (group) level for standards versus baseline, deviants versus baseline, and targets versus baseline, as well as creating MMN contrasts comparing blocks with deviants to standard only blocks for both stimulus combinations A and B.

### 2.8 ROI analysis

Regions of Interest were selected based on those identified across previous studies examining frontal sources of auditory mismatch [10]. Four separate masks were created for the left and right inferior temporal gyrus (IFG) and left and right middle frontal gyrus (MFG), as defined by the probabilistic Harvard-Oxford cortical structures atlas [12]. Activation within these regions was analysed using non-parametric permutation tests (RANDOMISE, http://www.fmrib.ox.ac.uk/fsl/randomise/, version 2.9) inference based on threshold-free cluster enhancement and FWE-corrected *p *< 0.05 (with default parameters *H* = 2, *E* = 0.5; [62]).

## 3 Results

### 3.1 Behavioral performance

The response rate to targets was greater than 99% for all participants in all conditions, so these were not analysed further. RTs were analysed with a 2 (imaging method) × 2 (stimulus combination) × 2 (block type) repeated-measures ANOVA. This revealed that participants were significantly faster in responding to targets in the MRI sessions (*M* = 398 ms, SD = 44 ms) than in the EEG session (*M *= 442 ms, SD = 88 ms), *F*(1,19) = 8.10, MSE = 2334.78, *p *= .01, *η*^2^_p_ = .299. Further, participants were faster to respond to targets in stimulus combination A (double-bar deviant, *M *= 416 ms, SD = 74 ms) relative to stimulus combination B (single-bar deviant, *M *= 424 ms, SD = 72 ms), *F* (1,19) = 5.03, MSE = 463.92, *p *= .037, *η*^2^_p_ = .209. No other main effects or interactions reached significance (all *p*s > .1). Critically for our interpretations of the imaging results, there were no significant behavioral differences associated with the presence of deviant stimuli in the blocks.

See Supplementary info A for a full description of the RTs.

### 3.2 Event-related potentials

**Fig. 2:**
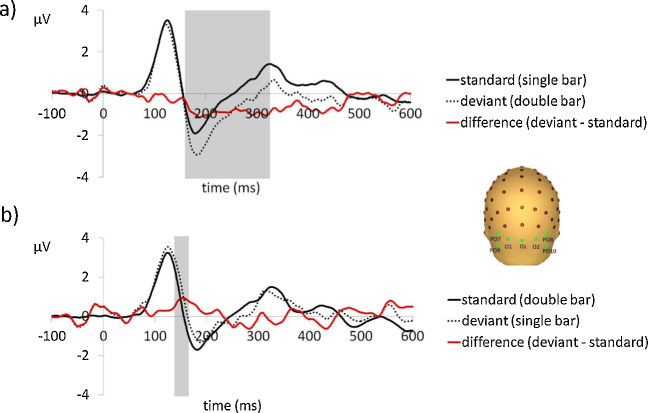
Grand average waveforms for a) stimulus combination A and b) stimulus combination B. The difference waveforms (i.e., deviant minus standard) are displayed in red. Values are based on the average voltage of the occipital region of interest electrodes (O1, Oz, O2, PO9, PO10, PO7 and PO8), indicated on the topographic figure. Shaded areas indicate epochs of significant difference (*p <* 0.05) between responses to standard and deviant stimuli.

Grand average waveforms for both stimulus combinations are presented in Fig. 2.

#### 3.2.1 Stimulus combination A

A clear vMMN was observed in the grand average waveforms. The response to standards and deviants differed significantly for a period of 169 ms between 161 and 329 ms post stimulus onset, as determined by sequential one sample *t*-tests using the Guthrie and Buchwald method [24]. The mean amplitude of the vMMN response during this epoch was −0.92 uV (±1.4).

#### 3.2.2 Stimulus combination B

No vMMN was observed in response to stimulus combination B. There was an early increased *positivity* in response to deviant stimuli lasting 32 ms from 138 to 170 ms, potentially due to a greater P1 response to deviant stimuli.

#### 3.2.3 vMMN validation

To further examine the differences in responses to stimulus combinations A and B and to ensure that the vMMN observed in stimulus combination A was not simply due to physical differences in the stimuli, an alternative subtraction method was used. Responses to single bars as *standard* stimuli were subtracted from responses to single bars when presented as *deviants* in a separate block. The same method was used to examine responses to double bar stimuli. Double bar deviants compared to double bar standards elicited a clear vMMN response for a period of 206 ms between 162 and 368 ms post stimulus onset, as determined by sequential one sample *t*-tests using the Guthrie and Buchwald method [24]. The mean amplitude of the vMMN response during this epoch was −0.94 uV (±1.7). Single bar deviants compared to single bar standards did not elicit a vMMN. The grand average waveforms for these comparisons are presented in Supplementary Info C. It is clear that the vMMN response observed in stimulus combination A was not due to physical differences in the stimuli and was the more robust combination for eliciting a vMMN response. The focus of the MRI analysis was therefore on responses to stimulus combination A.

### 3.3 fMRI: whole brain analysis

**Fig. 3:**
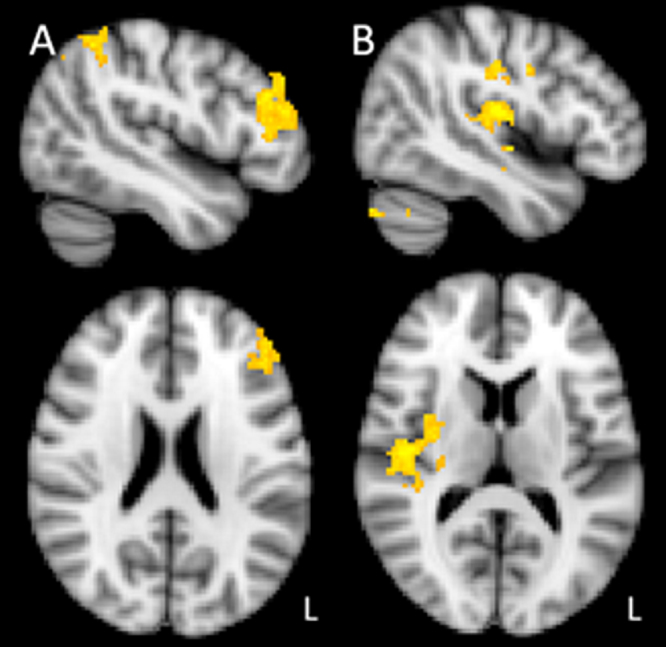
Regions of activation observed in whole brain analysis in stimulus combination A (single bar standards, double bar deviants). A (left side, MNI axial = 28, sagittal = 50) shows increased activity in deviant blocks relative to standards. B (right side, MNI axial = 14, sagittal = 46) shows increased activity in standard only blocks relative to blocks containing deviants. L indicates left hemisphere. Group statistics were calculated using a mixed effects general linear model with a cluster forming threshold Z > 2.3, and cluster corrected at *p* < 0.05.

We initially conducted a whole brain exploratory analysis, and significant clusters of activation reported in Table 1. Combination A (double-bar deviants) produced increased activity in the left MFG, IFG and frontal pole, when contrasting blocks containing deviants to standard-only blocks. Furthermore, this contrast revealed increased activation in left supramarginal gyrus, extending in to angular gyrus and lateral occipital cortex. In the reverse contrast, increased activity was observed in standard only blocks in right central opercular cortex (extending to the parietal operculum, insular cortex and Heschl’s gyrus), right postcentral gyrus, and right cerebellum. These regions of activation are shown in Fig. 3. In combination B, consisting of single bar deviants and double bars standards, increased activity to standard- only blocks was observed in a cluster extending through insular cortex, Heschl’s gyrus and central opercular cortex. No increases in activity were found when contrasting deviant blocks relative to standard-only blocks with this combination (B).

### 3.4 fMRI: region of interest analyses

**Fig. 4:**
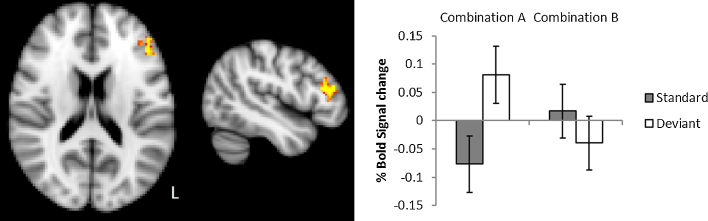
Left frontal activity observed in response to visual deviant stimuli, assessed using regions of interest and permutation testing (MNI axial = 16, sagittal = −46). Increases activity to double bar deviants (combination A) extended across left IFG, MFG and frontal pole. Statistical inference was performed using RANDOMISE permutation testing and activation assessed via threshold-free cluster enhancement within the left IFG mask (corrected *p *< 0.05). On the right, the bar plot shows the average percentage BOLD signal change (and standard error) relative to baseline for both stimuli combinations within the activated left frontal region, and indicates the relative difference between blocks containing deviants and the standard-only blocks.

The ROI analysis revealed significant clusters using both the left IFG and MFG masks, encompassing the same overlapping region. A 0.16% increase in BOLD activation was observed for blocks containing the combination of double-bar deviant stimuli and single-bar standards compared to standard only blocks (See Fig. 4). The cluster extended across the left IFG (29%), MFG (23%), and frontal pole (20%; peak activation in MNI (−46, 35, 14), *t* = 5.49, *p* = .0021). No other significant clusters of activation were indicated in any other regions or stimulus combinations

## 4 Discussion

We observed an increase in activation in the left IFG/MFG during blocks containing infrequent, ‘deviant’ stimuli, relative to blocks consisting only of ‘standard’ stimuli. Ours is the first functional imaging study, to our knowledge, to examine the neural mechanisms underlying the vMMN response without a concurrent attention demanding task (c.f., [82]). The involvement of frontal regions mirrors findings from the aMMN literature [10,33], and this correspondence corroborates suggestions for a multi-modal mechanism for the low-level detection of changes in the sensory environment [15]. While the current data provides evidence of a visual parallel to the aMMN frontal source, to establish the presence of a multi-modal frontal source future work should incorporate MMN paradigms across modalities, to allow direct comparison within subjects.

The role of the frontal mechanisms underlying the aMMN has been described as one of attention orientation or triggering, following from the detection of change in sensory processing regions [10,41]. The association of this function with the IFG in the literature is consistent with models implicating this region in alerting and executive attention networks (e.g., [17]), as well as proposals that sub-regions of the right inferior frontal cortex support the detection of a behaviorally relevant cue [79]. The observation of left, and absence of right, IFG activation in our study differs from the typical association with right IFG functioning in both the aMMN [10] and cognitive control literature (see [1], for a review); however, ours is also not the first study to observe activation in the left hemisphere in either of these domains (e.g., [39,73]). In both domains, there has yet to be a consensus regarding why left, or bilateral, IFG involvement is observed in some studies and not in others. One possibility is that activity in left IFG reflects in the way in which participants maintain task rules in response control tasks, either in an abstract or verbal form [4]. It is not clear how this interpretation might translate to discrepancies in the MMN literature, as the change itself is typically outside the focus of attention, and in the case of our task, the explicit rule (respond to the target) was constant in both standard only and deviant blocks. It is possible that the combination of a rare stimulus requiring a response (the target) with one that does not (the deviant) moderates the involvement of different regions. In line with this suggestion, studies examining the electrophysiological P3 response have demonstrated differing recruitment of frontal regions to task relevant versus task irrelevant events/stimuli (see [51] for a review). Our task is similar to the three stimulus oddball paradigm, which consists of standard, deviant and target stimuli. In this paradigm, the non-target deviants typically elicit a frontally generated P3a component, whereas target stimuli are likely to have elicited a frontally generated P3a and a parietally generated P3b. In contrast, the deviant stimuli in our paradigm generally do not elicit a clear frontal P3a, either in the current study (see Supplementary info B1) or in previous papers using identical stimuli [67–69]. We believe this to be due to the ease of discriminating our target stimuli from standards and deviants, which has previously been linked to significantly reduced or absent P3a responses [52]. Further, our target stimuli also elicited N2 and P3b ERPs reflecting an explicit attentional focus and response to these stimuli. These components were not present the in the deviant ERPs, confirming that deviant stimuli were not directly attended to. Thus, whilst the temporal resolution of fMRI generally make it difficult to distinguish activity associated with the MMN from later frontal components, we believe that it is unlikely that our observed activation is related to P3a activity. Nevertheless, future work with other MMN task variants should address the extent to which frontal sources reflect attentional capture and/or the potential suppression of attentional shifts to distracting stimuli.

vMMN was only elicited in response to stimulus combination A. To discount the possibility that the vMMN activity observed was a stimulus specific response, a follow up analysis on the ERP data was conducted. We verified that the response to the double bar stimulus observed in the combination which did elicit a vMMN (double bar deviant, single bar standard) differed relative to the response to a double bar when it was presented as a standard. In other words, the ERP effect reflected the context of the double bar (i.e., as a deviant), rather than the difference in physical properties of the stimuli. The observation that not all stimulus combinations are equally effective at eliciting a MMN response has also been made in the auditory domain, for example, increments in frequency were more effective than decrements [28]. Recent developments in the design of vMMN paradigms have offered more sophisticated ways of controlling for stimuli differences [54,61], however these paradigms would not be suitable for a block design fMRI study. The paradigm used in the current study was chosen to best fit the demands and constraints of both event related EEG and block design fMRI data acquisition. Whilst the absence of a vMMN response to stimulus combination B was neither anticipated nor desired, the concomitant absence of an increase in BOLD in the ROI analysis provides a useful dissociation, i.e., when a vMMN response is observed in the EEG data, it is accompanied by an increase in left frontal BOLD, however when a vMMN response is not observed there is also no increase in left frontal BOLD. Further, this paradigm has previously been successfully used to elicit vMMN across healthy younger adults, healthy older adults and dementia patients (see [75–77]). Thus, we are confident that the activity reported for combination A reflects the detection of change in the visual environment.

In addition to the hypothesised activation in the IFG in deviant blocks, we also observed an increase in activation in left supramarginal gyrus to blocks containing deviants relative to standard-only blocks. As with the left IFG, this region has typically been associated with linguistic processing [6,7] and verbal working memory [49], though may moderate response control via a process of semantic selection [73]. As suggested above, it is possible that activity in this region reflects the combination of a stimulus change that requires a motor response with one that does not in deviant blocks, i.e., responding to rare target stimuli, but not to rare deviant stimuli. We also observed an increase in activation in several regions in standard only blocks, relative to deviant blocks. These regions included right central opercular cortex, right postcentral gyrus, and right cerebellum. This increase was not predicted, and we can only speculate as to why some regions would show an increased activation in our task in the absence of deviant stimuli. The standard-only blocks still require participants to respond to targets, so participants are not passive or at rest during this time. An alternative explanation is that these regions support a mechanism that filters predictable, irrelevant information in standard-only conditions, and that this process is disrupted when deviant stimuli are present. It has been suggested that the cerebellum may play such a role in the aMMN [38].

The lack of correspondence between the effects observed from scalp EEG and fMRI in this task might at face value be viewed as problematic; however we believe emphasises the utility of using multiple measurement techniques. With respect to the absence of frontal effects in the ERPs in our data, the dipoles responsible for the early P1 and N1 ERPs are orientated in a manner that results in a voltage peak at occipital electrodes, and crucially the inverse voltage at frontal electrodes [13]. This makes it almost impossible to dissociate a frontal vMMN source, which may, like the frontal aMMN source be relatively weak, from the inverse of the P1 and N1 responses. This is not the case in the aMMN response in which the bilateral auditory dipoles result in a voltage maximum at the vertex, allowing for the easier identification and dissociation of frontal activity (e.g., [21]). Secondly, the orientation of the frontal generators are unknown and it is possible that they are orientated in the least optimum manner for detection at frontal electrode sites. For this reason, it is possible that fMRI techniques may be better suited to detect frontal activity associated with the detection of deviant stimuli that was unobservable (in this study) using EEG alone. Spatial modeling of the vMMN response using the anatomical co-ordinates provided in this paper provide a potential solution to this problem for future studies, i.e., a data driven a priori location in which to seed vMMN dipoles.

The absence of differences in activation in visual areas in our fMRI results contrasts to observed occipital generators of the vMMN that have been reported in previous EEG [33] and MEG [72]. In addition, the scalp distribution of the vMMN in our ERP findings centers over visual areas. We do not consider the absence of deviant related BOLD activity in occipital regions in our study to be evidence against the existence of occipital mismatch detectors. Rather, we suspect that the choice of a block design for our study resulted in insufficient power to detect the short-lived, transitory difference reflected in the 169 ms vMMN we observed, on top of the average of the activity common to both conditions. In contrast, we conceive of a frontal change detection mechanism that responds only to deviant stimuli, making BOLD responses more easily dissociable from any ongoing activity. Whilst our focus was to examine frontal mechanisms underlying the vMMN, future work may focus on the modulation of visual sensory regions, and how they interact. Our position is that the EEG may be insensitive to the frontal sources of the vMMN response, and that the MRI block design in the current study was optimised to detect frontal rather than occipital activation. In combination the techniques allow us to see what the other is missing, i.e., we believe that the vMMN response involves an interaction of both occipital and frontal regions (c.f., [31,65]).

In conclusion, our findings suggest that, like the aMMN, a frontal mechanism underlies change detection in the vMMN paradigm, in this case localised to the left IFG. This corresponds to observations of left IFG activation to deviant stimuli in the auditory modality, and raises the possibility of a common frontal change detection mechanism. The understanding of sensory change detection across modalities in the brain has significant implications for both theoretical and applied fields of cognitive neuroscience. MMN is emerging as a marker of cognitive decline across a range of diseases including schizophrenia and dementia [43], and identifying the structures responsible for MMN generation may help in the understanding and treatment of these conditions. In addition, the observation of IFG activation in association with the passive detection of visual change indicates that models of attention would benefit from a greater understanding of the role of inferior frontal cortex and its sub-regions in visual change detection.

## Figures and Tables

**Table 1:** Significant clusters of activation observed from whole brain analysis.

		MNI 152					
Contrast	Region	*x*	*y*	*z*	Voxels	*Z* score	*P*
Stimulus combination A: deviant > Standard	Left middle frontal gyrus	−50	28	28	612	3.72	<.001
	Left supramarginal gyrus	−48	−46	54	439	3.39	<.001
Stimulus combination A: standard > deviant	Right central opercular cortex	46	−18	14	762	3.74	<.001
	Right postcentral gyrus	38	−12	30	284	3.45	<.001
	Right cerebellum	36	−84	-32	380	3.73	<.001
Stimulus combination B: standard > deviant	Right insular cortex	42	−14	6	376	3.38	<.001

*Note*: Region, co-ordinates and *z* score reflect peak voxel of cluster. Regions identified from Harvard cortical atlas [12].
