# Supplementary material for: A frontal attention mechanism in the visual mismatch negativity
Source: Behav Brain Res. 2015 Oct 15;293:173–81. doi: 10.1016/j.bbr.2015.07.022 (PMC4566935; doi:10.1016/j.bbr.2015.07.022)
Supplement: Supplementary file 1 [file mmc1.pdf]

Supplementary Info A

Supplementary Table A1. Mean reaction times to targets for each condition and stimulus combination in the EEG and fMRI sessions. Standard deviations are shown in parentheses.

	EEG		fMRI	
	Combination A	Combination B	Combination A	Combination B
Standard only blocks	389ms (45)	401ms (43)	436ms (94)	449ms (94)
Deviant blocks	402ms (48)	399ms (41)	437ms (87)	446ms (83)

Supplementary Info B1

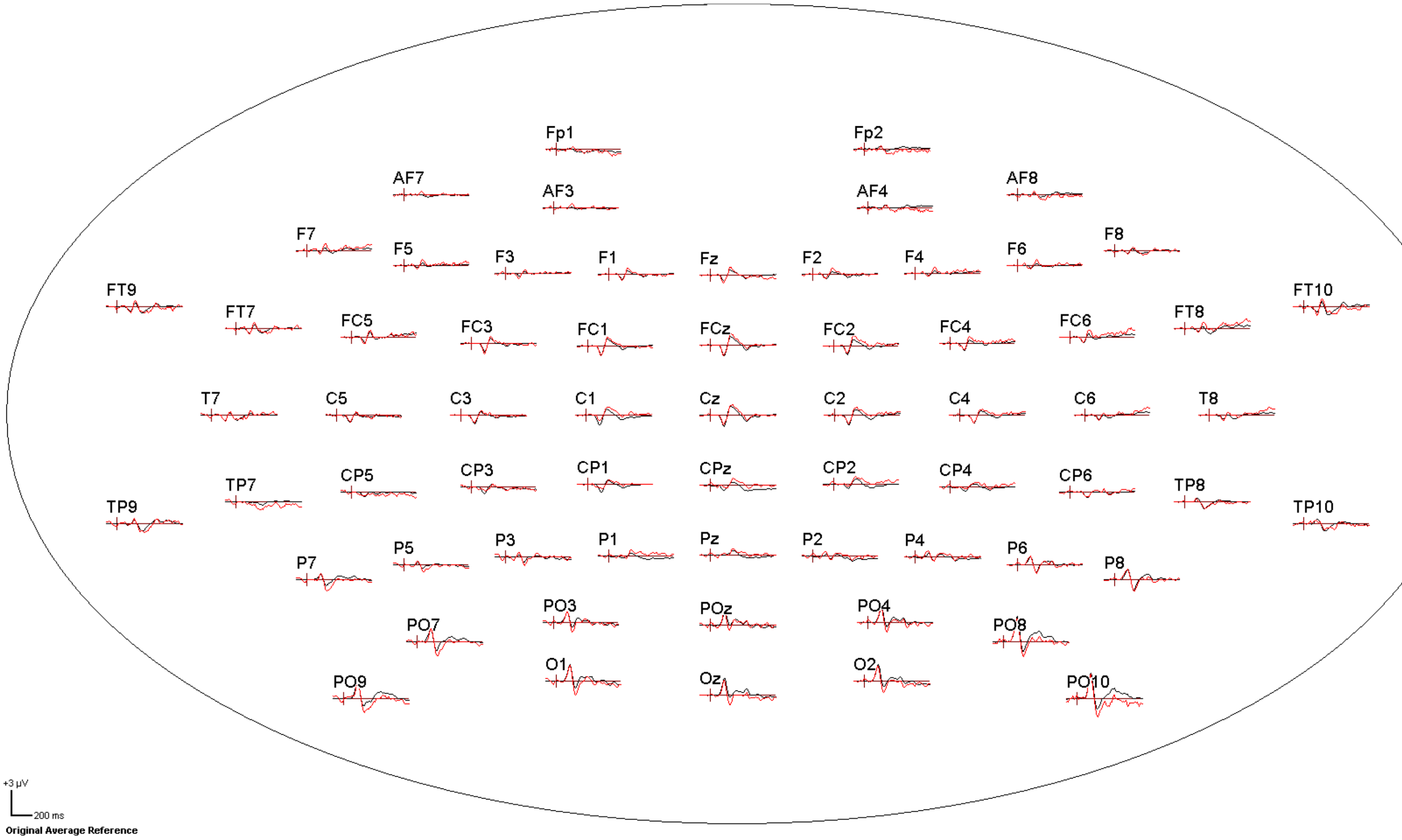

Supplementary Figure B1.64 electrode grand average plots of responses to standard (black) and deviant (red) stimuli for Stimuli combination A, referenced to a common average reference.

Supplementary Info B2

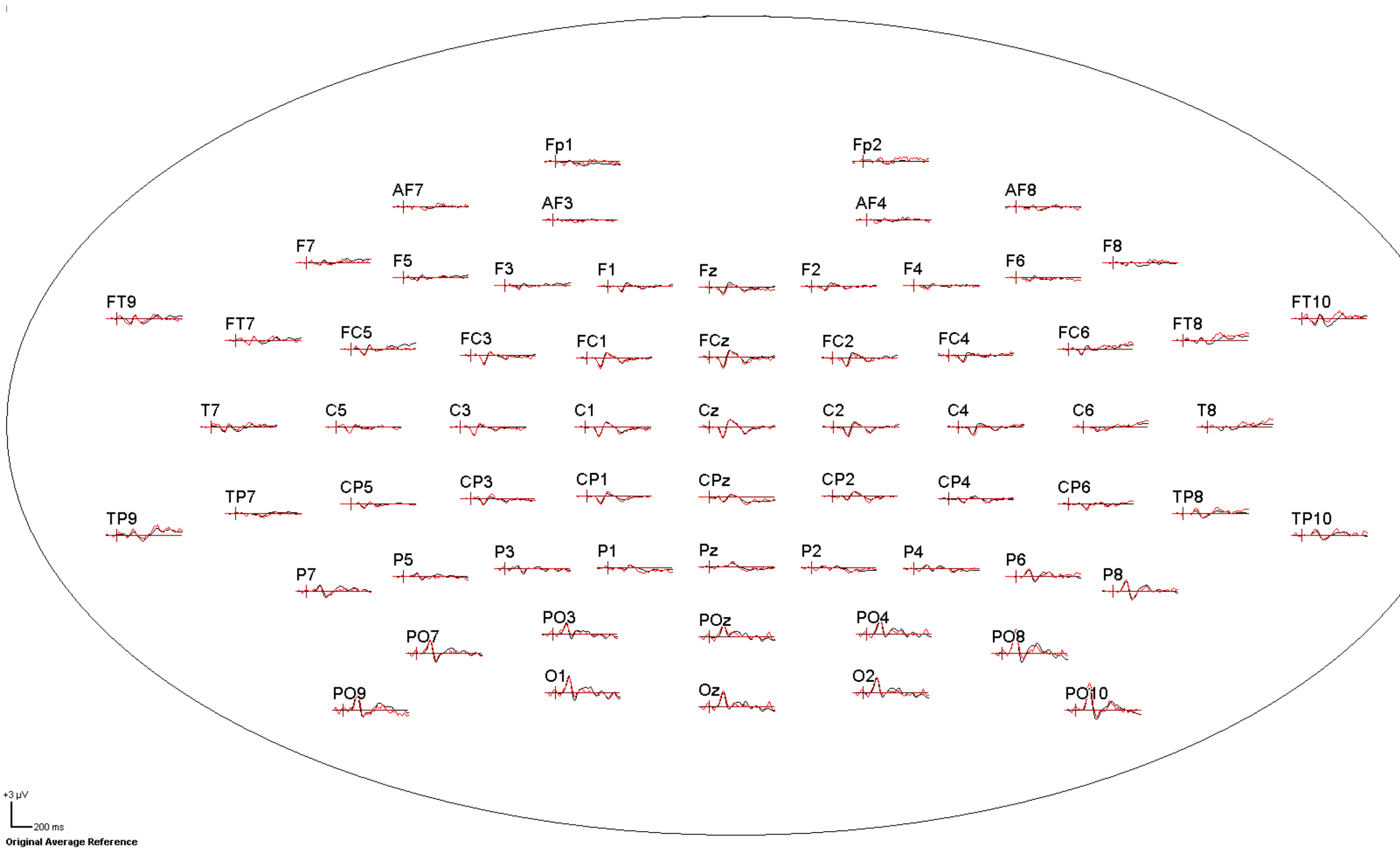

Supplementary Figure B2.64 electrode grand average plots of responses to standard (black) and deviant (red) stimuli for Stimuli combination B, referenced to a common average reference.

Supplementary Info C

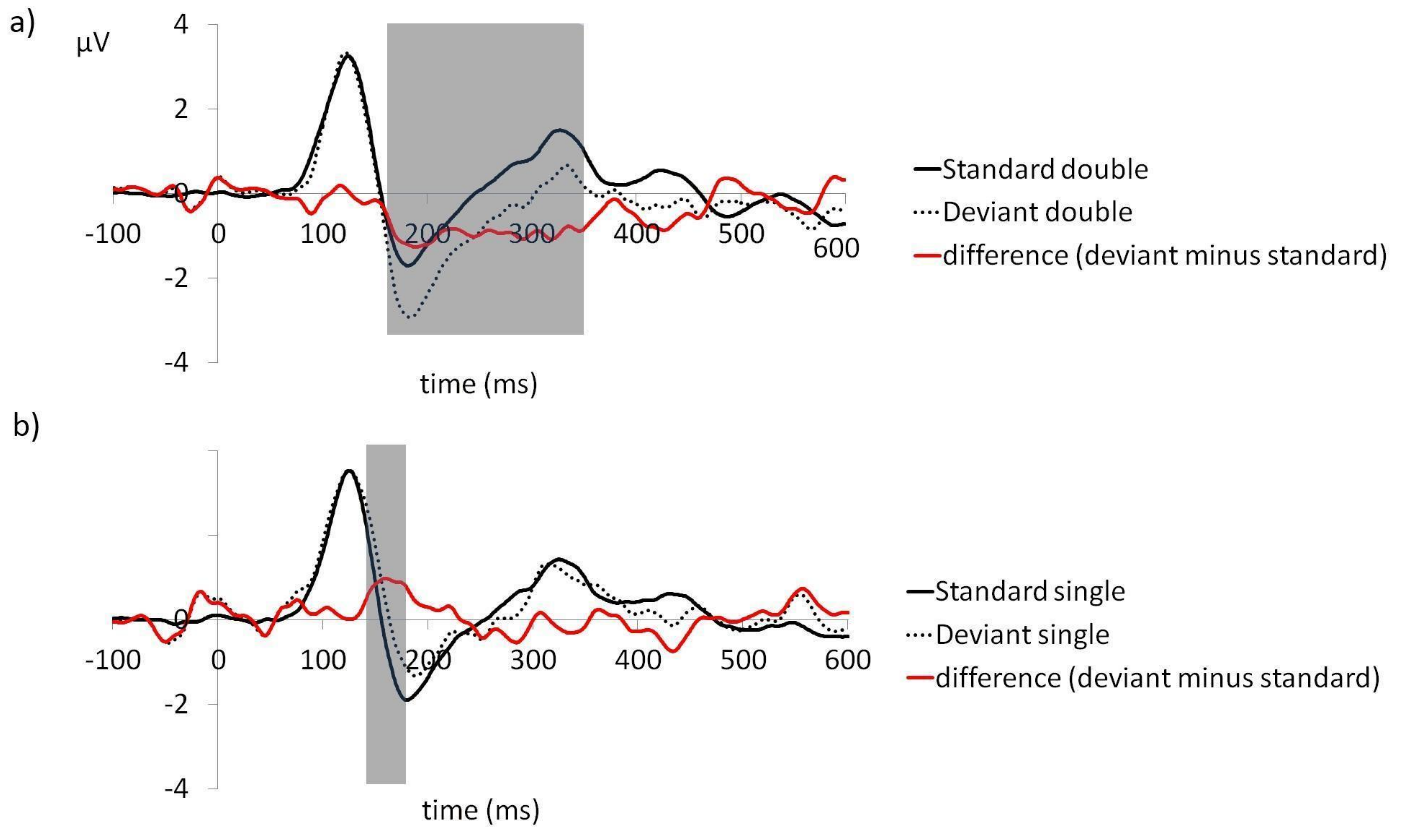

Supplementary Figure C. Grand average waveforms for a) Double bars as standards and deviants b) Single bars as standards and deviants. The difference waveforms (i.e. deviant minus standard) are displayed in red. Values are based on the average voltage of the occipital region of interest electrodes (O1, Oz, O2, PO9, PO10, PO7 & PO8). Shaded areas indicate epochs of significant difference ($p < 0.05$) between responses to standard and deviant stimuli

Supplementary Info D.

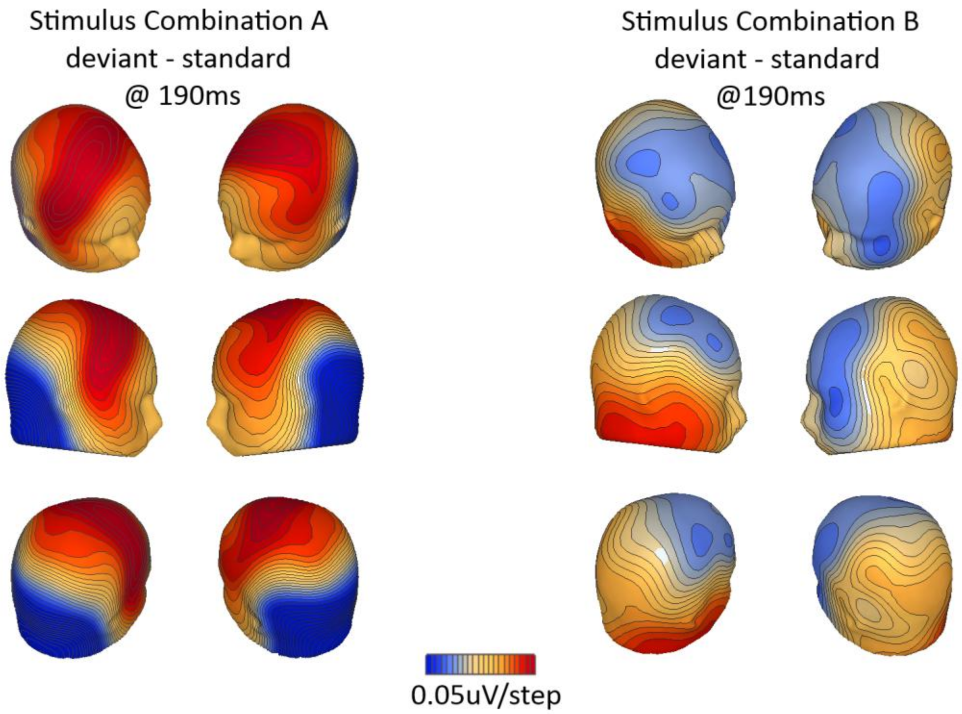

Supplementary Figure D. Topographic distribution illustrating the vMMN for Stimulus Combination A and the absence of vMMN for Stimulus Combination B. Plots indicate the difference in voltage distribution (deviant minus standard) across the scalp at the peak latency (190ms) of the vMMN in response to Stimulus Combination A. No vMMN was observed in response to Stimulus Combination B. Red indicates positive voltage, blue indicates negative voltage.
